# 

**Published:** 1875-11

**Authors:** 


VOL. 22. NOVEMBER, 1875. No. 11.

TWENTY-SECOND YEAR.

### HALL'S Journal of Health.

COPYRIGHT 1875.

PUBLISHED BY THE AMERICAN AND FOREIGN PUBLICATION CO., 137 EIGHTH ST., NEW YORK.

Four doors East of 756 Broadway.

**Agents for the Trade:**

AMERICAN NEWS COMPANY, 121 NASSAU STREET.
NEW YORK NEWS COMPANY, 18 BEEKMAN STREET.

**Terms, Two Dollars a Year.**

**SINGLE NUMBER, 20 CENTS.**

